# Multilaboratory Survey To Evaluate Salmonella Prevalence in Diarrheic and Nondiarrheic Dogs and Cats in the United States between 2012 and 2014

**DOI:** 10.1128/JCM.02137-16

**Published:** 2017-04-25

**Authors:** Renate Reimschuessel, Michael Grabenstein, Jake Guag, Sarah M. Nemser, Kyunghee Song, Junshan Qiu, Kristin A. Clothier, Barbara A. Byrne, Stanley L. Marks, Kyran Cadmus, Kristy Pabilonia, Susan Sanchez, Sreekumari Rajeev, Steve Ensley, Timothy S. Frana, Albert E. Jergens, Kimberly H. Chappell, Siddhartha Thakur, Beverly Byrum, Jing Cui, Yan Zhang, Matthew M. Erdman, Shelley C. Rankin, Russell Daly, Seema Das, Laura Ruesch, Sara D. Lawhon, Shuping Zhang, Timothy Baszler, Dubraska Diaz-Campos, Faye Hartmann, Ogi Okwumabua

**Affiliations:** a U.S. Food and Drug Administration, Center for Veterinary Medicine, Office of Research, Laurel, Maryland, USA; b U.S. Food and Drug Administration, Center for Veterinary Medicine, Office of New Animal Drug Evaluation, Rockville, Maryland, USA; c U.S. Food and Drug Administration, Center for Drug Evaluation and Research, Silver Spring, Maryland, USA; d California Animal Health and Food Safety Laboratory, University of California, Davis, Davis, California, USA; e School of Veterinary Medicine, University of California, Davis, Davis, California, USA; f College of Veterinary Medicine and Biomedical Sciences, Colorado State University, Fort Collins, Colorado, USA; g College of Veterinary Medicine, Department of Infectious Disease, Athens Veterinary Diagnostic Laboratory, The University of Georgia, Athens, Georgia, USA; h Veterinary Diagnostic and Investigational Laboratory, College of Veterinary Medicine, The University of Georgia, Tifton, Georgia, USA; i College of Veterinary Medicine, Veterinary Diagnostic Laboratory, Iowa State University, Ames, Iowa, USA; j College of Veterinary Medicine, North Carolina State University, Raleigh, North Carolina, USA; k Ohio Animal Disease Diagnostic Laboratory, Ohio Department of Agriculture, Reynoldsburg, Ohio, USA; l U.S. Department of Agriculture, National Veterinary Services Laboratories, Ames, Iowa, USA; m University of Pennsylvania, Matthew J. Ryan Veterinary Hospital, Philadelphia, Pennsylvania, USA; n Veterinary and Biomedical Sciences Department, Animal Disease Research and Diagnostic Laboratory, South Dakota State University, Brookings, South Dakota, USA; o College of Veterinary Medicine, Texas A&M University, College Station, Texas, USA; p Washington Animal Disease Diagnostic Laboratory, College of Veterinary Medicine, Washington State University, Pullman, Washington, USA; q Department of Pathobiological Sciences/WVDL, University of Wisconsin–Madison, Madison, Wisconsin, USA; University of Tennessee

**Keywords:** Salmonella, diarrhea, fecal organisms, pets, WGS

## Abstract

Eleven laboratories collaborated to determine the periodic prevalence of Salmonella in a population of dogs and cats in the United States visiting veterinary clinics. Fecal samples (2,965) solicited from 11 geographically dispersed veterinary testing laboratories were collected in 36 states between January 2012 and April 2014 and tested using a harmonized method. The overall study prevalence of Salmonella in cats (3 of 542) was <1%. The prevalence in dogs (60 of 2,422) was 2.5%. Diarrhea was present in only 55% of positive dogs; however, 3.8% of the all diarrheic dogs were positive, compared with 1.8% of the nondiarrheic dogs. Salmonella-positive dogs were significantly more likely to have consumed raw food (*P* = 0.01), to have consumed probiotics (*P* = 0.002), or to have been given antibiotics (*P* = 0.01). Rural dogs were also more likely to be Salmonella positive than urban (*P* = 0.002) or suburban (*P* = 0.001) dogs. In the 67 isolates, 27 unique serovars were identified, with three dogs having two serovars present. Antimicrobial susceptibility testing of 66 isolates revealed that only four of the isolates were resistant to one or more antibiotics. Additional characterization of the 66 isolates was done using pulsed-field gel electrophoresis and whole-genome sequencing (WGS). Sequence data compared well to resistance phenotypic data and were submitted to the National Center for Biotechnology Information (NCBI). This study suggests an overall decline in prevalence of Salmonella-positive dogs and cats over the last decades and identifies consumption of raw food as a major risk factor for Salmonella infection. Of note is that almost half of the Salmonella-positive animals were clinically nondiarrheic.

## INTRODUCTION

Salmonella infections are a serious cause of foodborne diseases in both humans and animals (1–4). In some cases, pet foods have caused human infections, presumably due to human handling of contaminated product (5–10). In the United States, between January 2012 and December 2015, there were more than 70 recalls of animal food or treats due to Salmonella contamination (http://www.fda.gov/Safety/Recalls/). It is unclear, however, what impact these contaminated animal feed products have on the occurrence of Salmonella infections in animals. In order to address this question, it is necessary to establish the background prevalence of Salmonella in the pet population.

Worldwide, a variety of surveys have been conducted to test for Salmonella in clinically healthy and diarrheic dogs (see Table S1 in the supplemental material). Data from 95 studies in 33 countries are included in Table S1. Fecal samples were taken from dogs under a variety of conditions, including those seen at clinics, in households, pet shops, shelters, laboratories, and an assortment of kennels, and working dogs, including therapy dogs and military dogs. Studies also varied greatly in isolation and detection methods. Prevalence, in nonoutbreak studies, ranged from 0 to 44%, with a median of 4% (average, 7.7%). In over a third of the studies, the prevalence of Salmonella-positive dogs was below 3%. Only six studies had prevalence estimates greater than 20%. Four of those investigations were conducted during or before the 1970s. Two of the studies were from more recent years. Leonard et al. (11) reported a high percentage of Salmonella-positive animals being fed raw diets. The remaining study with a 44% prevalence, by Jajere et al. in 2014 (12), was conducted in Nigeria. The authors of that study noted that the highest prevalence occurred in mongrel dogs, which were more likely to roam free, scavenge, and be fed raw food.

The studies conducted in the United States showed a general reduction in the frequency of Salmonella-positive animals in recent years. The median prevalence in all U.S. studies between 1949 and 2015 was 6.4% (average, 8.4%). The median prevalence for studies before 1980 was 8.7% (average, 10.6%), while studies after 1980 had a median of only 3.2% (average, 3.8%). Thus, it appears that in the United States, the prevalence of Salmonella in dog fecal samples declined approximately by half over the last 45 years. This general trend is similar to what is seen in studies from other countries. Overall, the median prevalence for other countries before 1980 was 7.2% (average, 8.5%), and after 1980, the median prevalence dropped to 3.5% (average, 6.3%).

These surveys suggest a lower prevalence of infection in dogs in the United States and also in many other countries in more recent years. This apparent decline in Salmonella infection prevalence occurred despite potentially improved methods for detecting the pathogen. It is important to remember, however, that the populations sampled and the laboratory techniques varied greatly between studies, and a definitive conclusion about prevalence based on a historical review is difficult to make.

Fewer studies have been conducted with samples from cats. Table S2 lists the Salmonella prevalence data in cats from 23 studies in 13 countries. Prevalences range from 0 to 13.6%, with a median of 2% (average, 3.9%). With cats, as with dogs, the median prevalence before 1980 was higher (3.9%) than that after 1980 (0.9%).

The U.S. surveys described above report data obtained primarily in a single state. There are no reports of studies conducted in multiple states during the same time period. The objective of the present study was to develop an estimate of the background prevalence of Salmonella in dogs and cats in the United States by testing animals presented at veterinary clinics. In the study we report here, 11 different diagnostic laboratories located in multiple sites across the United States cultured fecal samples from diarrheic and nondiarrheic dogs and cats for Salmonella by using harmonized methods. Participants in the study provided information on the animal's diet, current health status, exposure to other animals, and general demographic information. The results from 2,422 dogs and 542 cats included in this study are reported here.

## RESULTS

### Proficiency test of the V-CLASP method.

Results of the proficiency tests (PTs) using Salmonella enterica serovar Typhimurium isolated from dog fecal samples for the inoculum indicated that the laboratories using the Collaborative Laboratory Agreement Salmonella Project (V-CLASP) method performed as well or better than laboratories using other methods. V-CLASP laboratories met the acceptance criteria for the PT (ISO/IEC 17043:2010 [https://www.iso.org/obp/ui/#iso:std:iso-iec:17043:ed-1:v1:en]) with a success rate of over 90% in the PTs.

### Prevalence and risk factors.

A total of 2,964 animals, 2,422 dogs and 542 cats, were tested between January 2012 and April 2014 (Table 1). Only three cats were positive for Salmonella (prevalence, 0.06%). Of these, one was nondiarrheic, a 7-year-old female tuxedo with *S*. Javiana from North Carolina. Two cats had diarrhea, a 0.3-year-old neutered male Siberian with *S*. I 4,5,12:i:− from North Carolina and a 1-year-old male domestic shorthair tabby with *S*. Infantis from Ohio. All three cats were indoor and suburban. Only one cat ate raw food: the 0.3-year-old from North Carolina had been fed raw food by the breeder from 4 weeks until 14 weeks of age. The owner then switched the cat to a commercial diet. The owner also had given the cat a probiotic due to the diarrhea.

**TABLE 1:** Prevalence of Salmonella in fecal samples from diarrheic or nondiarrheic dogs and cats tested by laboratories located in 11 different states

State	No. of dogs	Total no. of Salmonella-positive dogs	No. of symptomatic dogs	No. of asymptomatic dogs	% Salmonella-positive dogs	No. of cats	Total no. of Salmonella-positive cats	No. of diarrheic cats	No. of nondiarrheic cats	Total no. of animals
CA	147	2	0	2	1	37	0			184
CO	274	4	2	2	2	49	0			323
GA	118	9	3	6	8	5	0			123
IA	288	5	1	4	2	88	0			376
NC	215	6	4	2	3	93	2	1	1	308
OH	144	5	5	0	4	6	1	1	0	150
PA	295	5	3	2	2	0	0			295
SD	424	7	4	3	2	93	0			517
TX	112^*a*^	11	5	4	10	20	0			132
WA	170	3	3	0	2	66	0			236
WI	235	3	1	2	1	85	0			320
All	2,422	60	31	27		542	3	2	1	2,964

a Two dogs had unknown diarrhea status.

Salmonella was isolated from 60 dogs (2.5%). Almost half (27/60, 45%) of the positive dogs were nondiarrheic, with 31 dogs (52%) having diarrhea at the time of sample collection. Clinical data for two positive dogs were not available. Of the 824 diarrheic dogs, 3.8% (13) were Salmonella positive, compared to 1.8% (14) Salmonella-positive nondiarrheic dogs. Thus, diarrheic dogs were more than twice as likely to be Salmonella positive than nondiarrheic dogs (Fisher's exact test, two-sided, *P* value = 0.005).

Of the dogs with diarrhea, 14 (45%) reported hemorrhagic diarrhea. Overall, the percentages of positive isolations from southern states, specifically Texas (10%) and Georgia (8%) (Table 1), were higher than the overall percentage of 2.5%. The percentages of positive dogs in the other states ranged between 1 and 4%.

### Temperature.

**FIG 1:**
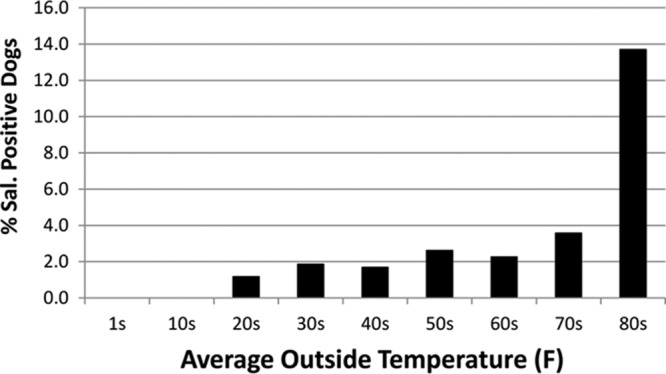
Percentage of Salmonella-positive isolates versus average monthly outside temperature of the state at the time each fecal sample was collected.

A higher percentage of Salmonella-positive dogs was identified when the temperatures were in the 80's (°F) (14%) than that at other temperatures, whose percentages ranged between 1 and ∼4% (Fig. 1). Since the temperature data were obtained from monthly averages, we did not conduct specific statistical tests on these data and are reporting only our observations.

### Dietary factors.

The main dietary factor that was associated with being positive for Salmonella was consumption of raw foods. Salmonella-positive dogs were more likely to have consumed raw food (16.7%, 10 of 60) compared to Salmonella-negative dogs (7.2%, 169 of 2,362) (Fisher's exact test, two-sided, *P* value = 0.01). Interestingly, feeding a probiotic, regardless of diarrhea status, was associated with Salmonella status. Salmonella-positive dogs were more likely to have consumed a probiotic than Salmonella-negative dogs (22.8% in Salmonella positive versus 9.3% in Salmonella negative; Fisher's exact test, two-sided, *P* value = 0.002). In addition, dogs fed raw food were more likely to have consumed a probiotic, regardless of current diarrhea status (Fisher's exact test, two-sided, *P* value of <0.0001 for no diarrhea and *P* value of 0.01 for diarrhea). Overall, 22.9% of dogs fed any raw food consumed a probiotic, while 8.5% of dogs fed no raw food consumed a probiotic. The multiple logistic regression analysis indicates that there is a statistically significant association (*P* = 0.03) between being positive for Salmonella and raw food consumption after controlling for probiotic consumption. Probiotics may be used as home remedies for diarrhea, and we evaluated the overall association of probiotic consumption with diarrheic versus nondiarrheic status. A history of diarrhea was positively associated with probiotic use at a *P* value of <0.0001.

Additionally, the multiple logistic regression analysis indicates that there is a statistically significant association (*P* = 0.004) between being positive for Salmonella and raw food consumption after controlling for residential area.

Other dietary factors (commercial treats, rawhide treats, chicken jerky, pig ears) did not show any significant statistical association with Salmonella status. More Salmonella-positive dogs (11.7%) than Salmonella-negative dogs (6.7%) consumed chicken jerky; however, there was no evidence of a statistically significant association between Salmonella status and chicken jerky consumption (Fisher's exact test, two-sided, *P* value = 0.19).

### Antibiotic use.

Of the 60 Salmonella-positive dogs, 20 had been treated with antibiotics (*P* = 0.01) There was also a statistically significant association between antibiotic use and diarrhea (*P* < 0.0001). Finally, there was a statistically significant association between antibiotic use and being positive for Salmonella when controlling for diarrhea (*P* = 0.047). The antibiotic most frequently used in the Salmonella-positive dogs was metronidazole.

### Age.

The age distribution of dogs sampled and percent positive are shown in Table 2. No evidence of a statistically significant association between being Salmonella positive and age was identified in this study, even for the <1-year-old age group versus those older than 1 year.

**TABLE 2:** Percentage of Salmonella-positive dogs by age

Age (yr)	% Salmonella-positive dogs vs all dogs in that age group	No. of Salmonella-positive dogs	Total no. of dogs
0–0.9	3	11	393
1–3	2	15	635
4–6	2	8	498
7–9	3	14	458
10–12	2	6	288
>13	3	4	117

### Housing and residential area.

For statistical analysis, we grouped dogs with any outdoor housing; i.e., dogs with both indoor and outdoor housing were grouped with dogs with outdoor-only housing. There was no evidence of a statistically significant association between Salmonella status and indoor housing versus any exposure to outdoor housing (Fisher's exact test, two-sided, *P* value = 0.15).

The residential area was associated with the Salmonella status. Urban dogs were less likely to be Salmonella positive than were rural dogs (odds ratio [OR] = 0.33; 95% confidence interval [CI] = 0.17, 0.66). Suburban dogs were less likely to be Salmonella positive than were rural dogs (OR = 0.37; 95% CI = 0.20, 0.66). However, there was no evidence of a statistically significant difference between urban dogs and suburban dogs (OR = 0.91; 95% CI = 0.46, 1.79).

### Exposure to other animals.

There was no evidence of a significant statistical association between the status of living with other animals in the same household and the Salmonella status. Exposure to livestock, including horses, overall did not show any evidence of a significant statistical association with the Salmonella status. We noted that more Salmonella-positive dogs than Salmonella-negative dogs were exposed to horses (11.7% versus 8.3%); however, there was no evidence of a statistically significant difference between positive dogs with no exposure to any livestock, including horses, and positive dogs with exposure to horses (OR = 1.56; 95% CI = 0.70, 3.49). There was also no evidence of a statistically significant association between being positive for Salmonella and attendance at shows.

### Hunting or sports status.

Neither activity, hunting nor sports, showed any significant statistical association with the Salmonella status.

### Exposure to water sources.

Surface water (exposed to untreated surface water, including ponds and streams) status did not show any significant statistical association with the Salmonella status.

### Isolate characterization: serovars.

Twenty-seven different Salmonella serovars were identified in the 64 isolates from the 60 positive dogs (Table 3). *S*. Newport was the most frequently isolated serovar (total, 13 isolates), followed by *S*. Enteritidis (total, 5), *S*. Javiana (total, 5), *S*. Typhimurium (total, 4), and *S*. Infantis (total, 5) (Table 3). Diarrhea was reported in at least one animal for all serovars except *S*. Braenderup, *S*. Cerro, *S*. Derby, *S*. Inverness, *S*. Mbandaka, *S*. Thompson, and *S*. Gaminara. Most of these serovars were isolated from only one animal, so we cannot draw any conclusions about whether these serovars are likely to cause diarrhea. *S*. Duval could not be recovered from the frozen sample following multiple attempts; only *S*. Mississippi grew from the archived samples. The National Veterinary Services Laboratories (NVSL) has observed this before when attempting to recover some serovars from a frozen mixed culture. Although the exact cause is uncertain, it is possible that this result is just indicative of an extremely low level of one serovar in relation to the others. Three dogs cultured positive for two different serovars. Of these, two dogs had two serovars isolated from the same fecal sample, while one dog had a repeat culture which isolated a different serovar from the first fecal culture. One of the original isolates (*S*. Albany) could not be recovered from the frozen archived sample and thus was not available for antimicrobial susceptibility testing (AST), pulsed-field gel electrophoresis (PFGE), or whole-genome sequencing (WGS) analysis.

**TABLE 3:** Serovars isolated from dog fecal samples between January 2012 and April 2014

Serovar	No. of isolates	State(s) of isolation (no. of isolates)	No. of dogs with diarrhea
*S*. Newport	13	GA (6), IA (2), NC (2), SD (2), TX (1)	3^*a*^
*S*. Javiana	5	GA (2), TX (2), NC (1)	2
*S*. Enteritidis^*b*^	5	SD (2), CA (1), CO (1), WA (1)	3
*S*. Infantis	4	OH (2), NC (1), TX (1)	3
*S*. Typhimurium^*b*^^,^^*c*^	4	CA (1), CO (1), IA (1), SD (1)	3
*S*. Anatum	3	TX (3)	3
*S*. Montevideo	3	OH (1), TX (1), WA (1)	2
*S*. Johannesburg	2	PA (1), WI (1)	1
*S*. Mbandaka	2	PA (2)	0
*S*. Paratyphi_B_var. _L-tartrate+^*d*^	2	OH (2)	2^*d*^
*S*. Worthington	2	CO (2)	2
*S*. I 4,5,12: i:−	2	IA (1), SD (1)	1
*S*. Albany^*e*^	2	WI (2)	1^*e*^
*S*. Berta	1	WA (1)	1
*S*. Braenderup	1	TX (1)	0
*S*. Carrau^*f*^	1	TX (1)	1
*S*. Cerro	1	SD (1)	0
*S*. Derby	1	IA (1)	0
*S*. Duval^*g*^	1	NC (1)	1^*g*^
*S*. Gaminara	1	TX (1)	0^*a*^
*S*. Give	1	NC (1)	1
*S*. Heidelberg	1	PA (1)	1
*S*. Livingstone^*d*^	1	OH (1)	1^*d*^
*S*. Mississippi^*g*^	1	NC (1)	1^*g*^
*S*. Schwarzengrund	1	PA (1)	1
*S*. Tallahassee^*e*^	1	WI (1)	1^*e*^
*S*. Thompson	1	CO (1)	0

a The diarrhea status of one dog was unknown.

b Two fecal samples were collected from the same dog, with different serovars in each fecal sample.

c *S*. Typhimurium and *S*. Typhimuriun var. O 5− were grouped together.

d Two serovars were isolated from the same fecal sample. Diarrhea was present in this dog and could be due to one or both of the serovars (*S*. Paratyphi_B_var._L-tartrate+ and *S*. Livingstone).

e One of the *S*. Albany isolates was in a mixed culture with *S*. Tallahassee but could not be reisolated for AST, WGS, or PFGE. Diarrhea was present in this dog and could be due to one or both of the serovars.

f Immunoserotyping first reported *S*. Madelia and then *S*. Carrau. WGS confirmed *S*. Carrau as the serovar.

g Two serovars were isolated from the same fecal sample. Diarrhea was present in this dog and could be due to one or both of the serovars (*S*. Duval and *S*. Mississippi).

The three positive cat isolates each had a different serovar, *S*. Javiana, *S*. I 4,5,12: i:−, and *S*. Infantis.

### AST.

Susceptibility testing showed that 60 (95%) of the 63 tested isolates from dogs and 2 of 3 isolates from cats were pansusceptible to the antibiotics tested (Table 4; Table S4). Only four isolates showed resistant phenotypes on the COMPAN2F panel. *S*. Typhimurium from California was resistant to chloramphenicol (>16 mg/liter), ticarcillin (>64 mg/liter), and ticarcillin-clavulanic acid constant 2 (>64 mg/liter). *S*. Derby from Iowa was resistant to doxycycline (>8 mg/liter) and ticarcillin (>64 mg/liter). *S*. Albany from Wisconsin was resistant to cefovecin (>4 mg/liter), cefoxitin (16 mg/liter), cefpodoxime (>16 mg/liter), ceftiofur (>4 mg/liter), ticarcillin (>64 mg/liter), and ticarcillin-clavulanic acid constant 2 (>64 mg/liter). This isolate had the highest MIC for cephalothin (>8 mg/liter) available on the panel, but we could not determine resistance, as the breakpoint for this antibiotic is ≥32 mg/liter. One isolate from a cat from North Carolina, *S*. I 4,5,12: i:−, was resistant to ticarcillin (>64 mg/liter). The results from the companion animal panel (COMPAN2F) and the NARMS Gram-negative panels were comparable.

**TABLE 4:** Comparison of susceptibility phenotype with that predicted by genotype^*a*^

Source of breakpoints^*b*^	Drug	Drug code(s)	Isolate D-01, *S*. Typhimurium			Isolate D-18, *S*. Derby			Isolate D-59, S. Albany			Isolate C-02, *S*. I 4,5,12: i:−		
			Genotype predicted by WGS	Phenotype based on COMPAN2F	Phenotype based on NARMs	Genotype predicted by WGS	Phenotype based on COMPAN2F	Phenotype based on NARMs	Genotype predicted by WGS	Phenotype based on COMPAN2F	Phenotype based on NARMs	Genotype predicted by WGS	Phenotype based on COMPAN2F	Phenotype based on NARMs
A	Amikacin	AMI	S	S	—	S	S	—	S	S	—	S	S	—
A	Amoxicillin-clavulanic acid (2:1 ratio)	AMC, AUG2	S	NI	S	S	NI	S	R	NI	R	S	NI	S
A	Ampicillin	AMP	R	NI	R	R	NI	R	R	NI	R	R	NI	R
D	Azithromycin	AZI	S	—	S	S	—	S	S	—	S	S	—	S
A	Cefazolin	FAZ	S	S	—	S	S	—	R	NI	—	S	S	—
E	Cefovecin	FOV	S	S	—	S	S	—	R	R	—	S	S	—
C	Cefoxitin	FOX	S	S	S	S	S	S	R	R	R	S	S	S
C	Cefpodoxime	POD	S	S	—	S	S	—	R	R	—	S	S	—
C	Ceftriaxone	AXO	S	—	S	S	—	S	R	—	R	S	—	S
D	Ceftiofur	XNL, TIO	S	S	S	S	S	S	R	R	R	S	S	S
A	Cephalothin	CEP	S	S	—	S	S	—	S	NI	—	S	S	—
A	Chloramphenicol	CHL	R	R	R	S	S	S	S	S	S	S	S	S
C	Ciprofloxacin	CIP	S	—	S	S	—	S	S	—	S	S	—	S
C	Doxycycline	DOX	S	S	—	R	R	—	S	S	—	S	S	—
A	Enrofloxacin	ENRO	S	S	—	S	S	—	S	S	—	S	S	—
A	Gentamicin	GEN	S	S	S	S	S	S	S	S	S	S	S	S
A	Imipenem	IMI	S	S	—	S	S	—	S	S	—	S	S	—
C	Kanamycin	KAN	R	—	R	R	—	R	S	—	—	S	—	—
A	Marbofloxacin	MAR	S	S	—	S	S	—	S	S	—	S	S	—
C	Nalidixic acid	NAL	S	—	S	S	—	S	S	—	S	S	—	S
D	Streptomycin	STR	R	—	R	R	—	R	S	—	S	R	—	R
A	Sulfisoxazole	FIS	R	—	R	R	—	R	S	—	S	S	—	S
A	Tetracycline	TET	R	—	R	R	—	R	S	—	S	S	—	S
C	Ticarcillin	TIC	R	R	—	R	R	—	R	R	—	R	R	—
B	Ticarcillin-clavulanic acid constant 2	TIM2	S^*c*^	R^*c*^	—	S	S	—	R	R	—	S	S	—
A	Trimethoprim-sulfamethoxazole	SXT, COT	S	S	S	S	S	S	S	S	S	S	S	S

a S, susceptible to drug; R, resistant to drug; NI, no interpretation (panel's highest drug concentration was lower than drug's breakpoint); –, drug not available on that plate.

b Breakpoints from the following sources were used for interpretation: CLSI M31-A3 (108) (A), CLSI VET01-S2E (109) (B), CLSI M100-S24 (110) (C), NARMS (114) (D), and EMA (115) (E).

c Discrepancy between phenotype and predicted genotype.

### PFGE.

**FIG 2:**
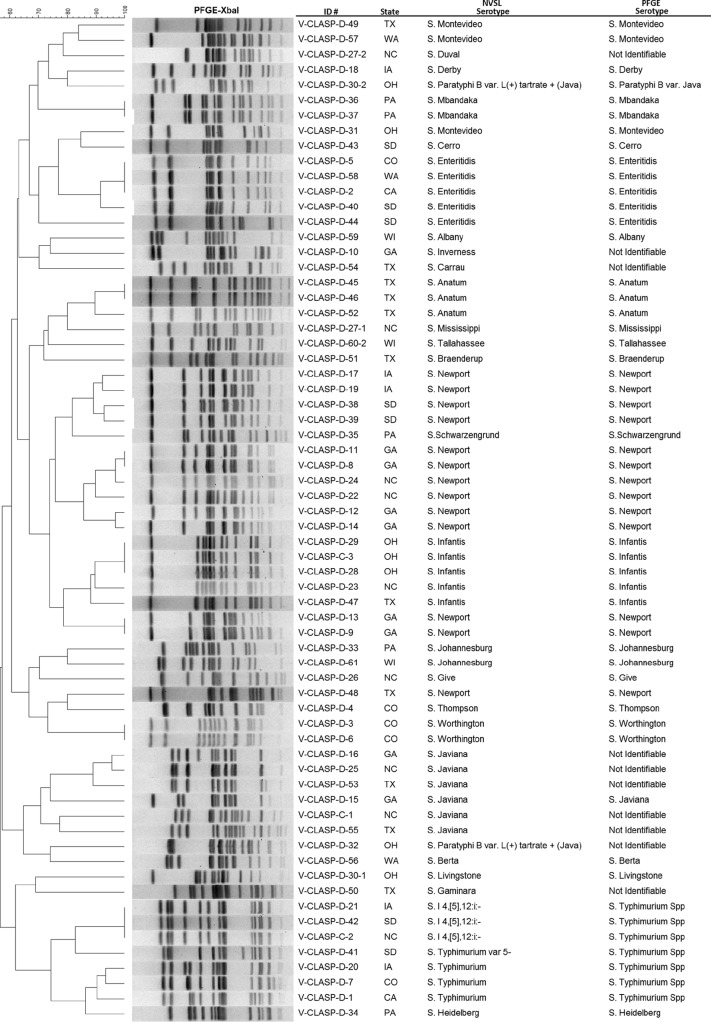
Dendrogram of Salmonella PFGE pulsotypes from dog and cat fecal samples collected by 11 laboratories in the United States from January 2012 through April 2014. Serovars determined by the National Veterinary Services Laboratories (NVSL) are compared with those predicted by PFGE.

PFGE analysis revealed 55 distinct pulsotypes from 66 isolates, 63 from dogs and 3 from cats (Fig. 2, dendrogram). The PFGE patterns were sent to the Centers for Disease Control and Prevention (CDC) PulseNet database to be compared with previous entries in PulseNet. Of the 55 pulsotypes, 46 (84%) patterns were present in the database, and 9 (16%) were new to PulseNet.

### WGS.

Antigenic serotyping compared well with immune grouping and PFGE as determined by SeqSero (15). Resistance genotypes correlated almost perfectly with phenotypes, as genes conferring resistance to beta-lactamases, streptomycin, kanamycin, chloramphenicol, tetracycline, and sulfisoxazole were all found only in isolates with resistance to these antimicrobial agents. These resistance genes were identical to some of those that have previously been identified in human strains of Salmonella (16). No resistance genes were identified in any of the pansusceptible isolates. The only case of a discrepancy between resistance genotypes and phenotypes was with the *S*. Typhimurium isolate from California, which displayed phenotypic resistance to ticarcillin-clavulanic acid. However, this isolate did have a *bla*_CARB-2_ gene that is expected to confer elevated MICs to this antibiotic but does not typically reach the breakpoint. Thus, overall resistance genotypes and phenotypes correlated in more than 99% of cases, as has been observed in previous studies (16).

WGS data also revealed information about the relatedness of the isolates to those previously sequenced and submitted to GenBank. Although most isolates were not found to be highly related to previous submissions, some isolates were phylogenetically close to isolates from humans or other sources. For instance, D-44 was an *S*. Enteritidis isolate from a dog in South Dakota and had only four single-nucleotide polymorphisms (SNPs) compared to a recent human PulseNet submission (Table 4). Isolates related to *S*. Infantis, *S*. Worthington, and *S*. Livingstone were also identified from pet food sources, suggesting that pet food may have been a potential source of the Salmonella bacteria colonizing the companion animals. The *S*. Infantis isolate in our study was isolated in the outbreak investigation reported by Imanishi et al. in 2014 (7).

### PCR comparison.

Six different PCR methods were compared. Five laboratories used “in-house” methods, while four laboratories used the MicroSEQ kits to test the archived buffered peptone water (BPW) samples. The data obtained from the MicroSEQ method and one of the in-house methods (method A) were the most consistently correct. This round of testing was conducted more than a year after the isolates had been obtained. Of the 34 previously cultured Salmonella-positive samples, only 13 could be reisolated from the archived BPW samples. These were correctly identified by most of the PCR methods. Of the 21 remaining samples from which Salmonella could no longer be isolated, 12 were correctly identified as positive by all the laboratories using the MicroSEQ method and the laboratory using method A. False positives rarely occurred when these two methods were used.

## DISCUSSION

This study provides information on targeted and periodic Salmonella prevalence in dog and cat fecal samples studied in multiple states by 11 laboratories using a harmonized method. Previous reports focused only on one state or on data collected over several years by a single laboratory. In addition to harmonization of the culture method, study participants also used the same case definition and a standardized questionnaire for feeding history and description of clinical presentation. It is important to remember when comparing the results of the present study with past reports that variations in the source of the animals sampled, i.e., stray or household, the season that samples are collected, the method of sampling the feces, i.e., swab versus 1-g samples, the culture methods, and the serotyping methods all can affect the results. Tables S1 and S2 in the supplemental material provide an extensive literature review with additional details about the various surveys that are discussed in this paper.

In the present study, we found an overall study prevalence of 2.5% of Salmonella in dog fecal samples collected between 2012 and 2014 by 11 laboratories located throughout the United States. The study prevalence in cats was only 0.06%. This reduced prevalence could be due, in part, to a smaller number of fecal samples being obtained from cats than from dogs. This is consistent with most other surveys worldwide that included both cats and dogs. Fewer samples have been collected from cats, and there has been a lower prevalence reported in cats (13, 14, 17–26). Only one study, that of Cruickshank and Smith in 1949 (27), reported a higher prevalence of Salmonella in feces of cats, 1.4%, compared to 1.0% for dogs. In that study, equal numbers of samples (500 each) were obtained from cats and dogs. As was mentioned before, most recent studies have lower prevalence estimates than studies prior to 1980. Since culture methods have improved over time, as described by Gorham and Garner in 1951 (17) and by Borland in 1975 (28), the present study would be expected to have detected higher numbers than those of some of the past studies if the actual prevalences were the same.

### Diarrhea.

The significance of diarrhea in the clinical history of cats and dogs was evaluated in this study. Two of the three cats (66%) that were Salmonella positive had diarrhea. Shimi and Barin in 1977 (29) also reported that diarrhea was more prevalent in the Salmonella-positive population, and Van Immerseel et al. in 2004 (30) noted that cats that were immunocompromised from other diseases were also more likely to be positive. Additionally, a nondiarrheic carrier state in cats has been demonstrated in multiple studies (31).

More Salmonella-positive than Salmonella-negative dogs in our study had diarrhea (55%), but even so, nearly half of the dogs were nondiarrheic. This is consistent with previous reports in dogs (24, 32–39). Indeed, in multiple Salmonella feeding experiments, nondiarrheic shedding in feces has been documented (38, 40–42). Similar results have been found in outbreak investigations in which diarrhea occurred in some of the animals but nondiarrheic animals were found positive upon testing (43, 44). Some of the experimental infection studies have also reported sporadic shedding for prolonged periods in nondiarrheic animals. It is therefore prudent to test repeated follow-up samples from the same animal if Salmonella has been previously diagnosed. Additionally, during a Salmonella outbreak or known exposure, screening nondiarrheic animals may provide a more accurate indication of prevalence.

### Distribution in the United States and temperature effects.

Overall, our results indicate that Texas and Georgia had higher prevalences of Salmonella-positive dogs than did the other states participating in the study. A greater prevalence estimate in the southern United States in dogs is consistent with what has been reported in other species, including humans. The reason for this is not known, but some have speculated that temperature may be a factor (45). Temperature may alter animal scavenging behavior and also the availability of clean water. In one African study, the authors found more cases during midsummer and early winter, noting scavenging behavior and increased rainfall at those times (46). Salmonella has been reported to be less prevalent in wild mice during winter (47). Multiple studies have reported increased Salmonella cases in humans during summer months (48–52). Ravel et al. in 2010 (50) indicated that in Canada, the prevalence of human salmonellosis over 4 years (2005 to 2008) increased in the summer months (June and July). The risk factors associated with this rise were gardening and attending barbeques, while the prevalence of Salmonella-positive retail poultry remained the same. The authors concluded that the product contamination rate did not vary with season but that the undercooking of meat during the summer months may have been responsible for the seasonal variability. Other studies in Germany, Netherlands, England and Wales, Switzerland, Spain, Czech Republic, Denmark, and Australia also indicated a relationship between outdoor temperature and Salmonella illness in humans (52–55). Improper food handling and increased bacterial growth in warm weather were considered potential factors that could contribute to the increased caseload. Notably, indirect effects, such as changing eating preferences by eating barbeque in hot months with a subsequent increased potential for consuming undercooked meat, were considered prime risk factors in all of these studies. Since companion animals may also be fed undercooked products or products that have increased bacterial growth in warm weather, it is not surprising that trends in Salmonella prevalence in dogs parallel those of the human population with respect to prevalence in southern U.S. states or environmental temperature.

### Dietary risk factors.

Salmonella-contaminated foods have been recognized as a risk factor in human and pet infections (1–4, 56). As mentioned before, in some cases the same product caused illness in humans and pets (7, 57). One of the most frequently reported risk factors for developing salmonellosis is the feeding of raw food to dogs (11, 58–60). Disease outbreaks in dogs have also been traced to contaminated raw product (44, 61–63). Experimental feeding studies have also demonstrated that a certain percentage of dogs fed Salmonella-contaminated raw foods can develop a carrier state, shedding the organism in their feces (40, 41). The present study also found a strong association between feeding raw food and the presence of Salmonella in fecal samples. In addition, Salmonella-positive dogs were more likely to have consumed a probiotic than Salmonella-negative dogs. Probiotic use was also associated with dogs having diarrhea. This is not surprising, as probiotics may be used to treat diarrhea. An association of Salmonella with probiotic consumption was also reported by Lenz et al. in 2009 (60) and by Leonard et al. (11). In our study, dogs fed raw diets were also more likely to have been fed a probiotic, regardless of current diarrhea status. It is unclear at this point if probiotics are more commonly used by owners due to a preference for a certain type of diet. Proponents of “natural” or raw diets may be more likely to also try “natural or home” remedies or supplements. A statistically significant association does not necessarily indicate a causal relationship. We are not aware of any studies that have tested probiotics for the presence of Salmonella. Since this is not the first time that there has been a potential association between probiotic use and Salmonella infection in dogs, further studies to understand this association may be warranted.

Studies examining dog foods for Salmonella show that over time, occurrence of Salmonella-positive commercial dry diets has declined over the years, very likely due to better process controls (64–67). There are, however, ample studies showing that raw diets still culture positive in surveys of dog food products (68–70) or when linked to outbreaks (43, 71). Dog treats have also tested positive in past surveys (72; https://www.fda.gov/AnimalVeterinary/ResourcesforYou/AnimalHealthLiteracy/ucm278271.htm); however, treat consumption did not show any evidence of a significant statistical association with the Salmonella status in our study. More Salmonella-positive than -negative dogs consumed chicken jerky (11.7% versus 6.7%), but there was no evidence of statistical significance.

### Other risk factors. (i) Antibiotic use.

We found that there was a statistically significant association between antibiotic use and diarrhea. Additionally, there was a statistically significant association between being positive for Salmonella and antibiotic use. Of the 20 Salmonella-positive dogs, 8 had been treated with the antibiotic metronidazole, which may be used to treat chronic diarrhea and protozoal intestinal infections. It is not clear if the antibiotics were associated with positive Salmonella results due to an effect on the gut bacterial populations, if the association with diarrhea and antibiotic use was a contributing factor, or if both of these factors were involved. This is an area which should be explored further, as it may inform clinicians regarding best practices for treating dogs that are infected with Salmonella.

### (ii) Age.

Data from our study did not find an association between age and being Salmonella positive. This is consistent with several surveys in dogs (32, 39, 45, 73). Other studies (42, 46, 58, 74–77) have reported younger and older animals as having a greater likelihood of serious infection. In humans, the young and the elderly are most at risk for developing invasive salmonellosis (78). None of our positive cases reported serious invasive infections.

### (iii) Housing and residential area.

Initially, dogs were categorized into three groups: indoor, outdoor, and both indoor and outdoor. The indoor/outdoor status was associated with the Salmonella status (outdoor dogs being more likely to be Salmonella positive) when dogs with both indoor and outdoor housing were excluded from the analysis (Fisher's exact test, two-sided, *P* value = 0.02). If, however, dogs with both indoor and outdoor housing were added to the outdoor-only group, there was no evidence of a statistically significant association between the Salmonella status and indoor dogs versus dogs with any outdoor housing status (Fisher's exact test, two-sided, *P* value = 0.15).

With respect to residence location, rural dogs were more likely to be Salmonella positive than either urban or suburban dogs. This is contrary to what was found in three other studies in which urban dogs had higher Salmonella prevalences than nonurban dogs (17, 37, 79). These studies, all from 1970 and earlier, included stray dogs, while our study included only household dogs. Indeed, Sakazaki et al. (79) indicated that there was almost zero occurrence of Salmonella in “rural dogs and house dogs even in a large city” in comparison with stray dogs.

### (iv) Livestock and other exposures.

Our study found no evidence of statistically significant differences in the Salmonella status of dogs living with other animals, including livestock. Hunting, attending sports events, or exposure to surface water, including ponds and streams, did not show any evidence of a significant statistical association with the Salmonella status. Some studies have shown that racing dogs have a high prevalence of Salmonella (80–82). This high prevalence, however, has been attributed more to the fact that many of these animals are given raw food, frequently contaminated with Salmonella (43, 68, 83). In the present study, raw food, not racing or outside activities, was associated with positive Salmonella status.

### (v) Sex.

In our study, we did not note any evidence of a difference in Salmonella prevalence between males and female dogs (Fisher's exact test, two-sided, *P* value = 0.36). Jajere et al. in 2014 (12) reported that in Nigerian stray dogs, males had a higher Salmonella prevalence but that almost twice as many samples from male dogs were tested and the results could be an artifact of sampling. No sex prevalence was identified in other studies (32, 39, 45, 73).

### Characteristics of the Salmonella isolates. (i) Serovars.

Many Salmonella serovars have been reported in dogs and cats (80, 84–86). The occurrence of various serovars has varied over time and geographic region. Some studies in various countries have noted that the serovars found more frequently in dog feces tend to be the same found in humans (42, 56, 59, 73, 80, 86, 87). Indeed, Sakazaki et al. in 1959 (79) indicated that yearly Salmonella serovar prevalence and geographic distribution changes observed in dogs were similar to those of humans. This report also described the occurrence of specific serovars following World War II but which were isolated from humans and dogs after the war, presumably due to military movement of personnel and perhaps animals.

In our study, a total of 27 unique serovars were identified from the 66 isolates. The top seven serovars found in dogs matched the top seven serovars (in a slightly different order) reported by the CDC in humans in 2012 (88) (Table 5). It is not surprising that the top seven serovars reported in humans and dogs are similar. As discussed previously, a common exposure route consisting of contaminated food, possibly raw or undercooked, likely accounts for many of the reported cases. In some cases, concurrent infections have been documented within the same household. The 2012 Salmonella Infantis outbreak in the United States is such a case (7). The common source was contaminated dog food, causing illness in dogs and humans in the same family. Several dogs in the present study were identified as positive for *S*. Infantis during the outbreak. In fact, the U.S. Food and Drug Administration (FDA) assisted the CDC's investigation by providing testing for animals in households with positive human cases. Having a harmonized method used by multiple laboratories around the country facilitated the public health investigation during the outbreak.

**TABLE 5:** Comparison ranking of top six serovars found in dogs with the same serovars in humans

Serovar	Rank order of serovar by:	
	V-CLASP method in dogs^*a*^	CDC in humans in 2012^*b*^
*S*. Newport	1	3
*S*. Javiana	2	4
*S*. Enteritidis	3	1
*S*. Infantis	4	7
*S*. Typhimurium	5	2
*S*. Montevideo	6	6

a Rank order of the serovars found in dogs, with the highest frequency indicated by 1.

b Ranking of the same serovars as found in humans, with the highest frequency indicated by 1 (list is in the order corresponding to the dog rankings).

### (ii) Antimicrobial susceptibility.

Of the 66 isolates in the present study, 62 were pansusceptible to the antibiotics tested (Table 4; Table S4). This is similar to the findings of many other surveys (32, 44, 45, 84, 85, 89–91). Two of the isolates in the present study were resistant to three or more antibiotics. Other authors, in multiple countries, have reported multiclass resistance in the Salmonella isolates from dogs (33, 73, 87, 90, 92). It is important to note that one case of resistance to colistin was identified in a dog in India (92). Colistin is considered a “last-resort” drug, and the *mcr-1* colistin resistance gene was recently reported in an isolate from a human case in the United States (93–96). The development of resistance in Salmonella is of concern, as it is one of the most frequently reported food safety pathogens. Efforts to reduce the development of resistant bacteria, such as the judicious use of antimicrobials in animals and humans, are important to protect public health.

### (iii) PFGE and WGS.

PFGE revealed 55 distinct pulsotypes in the 66 isolates examined. Of the 55 V-CLASP PFGE patterns, 27 patterns matched those associated with human isolates, involving a total of 207 outbreaks. Many of the patterns were associated with multiple outbreaks. Those serotypes involved in more than 10 outbreaks were also the ones frequently reported in human infections, namely, *S*. Typhimurium, *S*. Heidelberg, *S*. Enteritidis, *S*. Infantis, and *S*. Newport. Two of the outbreaks previously listed in PulseNet were identified as having a possible pet food source. Given the close contact that pets and families share, it is not surprising that some of the Salmonella PFGE patterns found in humans have now also been identified in dogs.

Antigenic serotyping was compared to PFGE and WGS groupings. All methods showed comparable results, except for the D-54 isolate. In this case, WGS reported *S*. Carrau. Serotyping initially indicated *S*. Madelia, but a repeat evaluation indicated *S*. Carrau. PFGE results were undeterminable. Such discrepancies have been noted previously for these two serovars (15). In addition, WGS identification of resistance genes correlated with the phenotypes obtained by routine antimicrobial susceptibility testing. There are few studies reporting PFGE profiles from dog Salmonella isolates (30, 43–45, 62, 97, 98). WGS data for dog Salmonella isolates have, at this point, been generally unreported. The results from the present study have been entered into a publicly available database (National Center for Biotechnology Information [NCBI] BioProject PRJNA314600) to facilitate comparisons between future studies and to facilitate outbreak investigations.

### Public health impact.

Salmonellosis is a major zoonotic disease, for which there have been many historical reports indicating transmission of the organism in food and farm animals but also from companion animals. Multiple case reports describing infections with transmission of the same isolate to humans and animals within a household have been published (7, 12, 21, 99–103) (https://stacks.cdc.gov/view/cdc/1723). The transmission pathway is not always clear but can involve transmission from food to animal, or food to human, or transmission between humans and animals. It is thus important to evaluate the risk factors that increase the likelihood of infection.

As has been seen with recent recalls, Salmonella-contaminated pet food products can be widely distributed and infect animals and their owners. In the United States, the estimated number of pets in 2012 was approximately 144 million, consisting of approximately 70 million dogs and 74 million cats. On average, over 50% of households owned a pet, 36.5% of households owned at least one dog, and 30.4% of households owned at least one cat in 2012. This information is available on the AVMA website (https://www.avma.org/KB/Resources/Statistics/Pages/Market-research-statistics-US-pet-ownership.aspx) and the HSUS website (http://www.humanesociety.org/issues/pet_overpopulation/facts/pet_ownership_statistics.html). It is therefore important to understand the potential for zoonotic disease transmission within a household. In order to address this issue, it is necessary to establish the background prevalence of Salmonella in the animal population. The present study obtained data from dogs and cats in 36 states during an approximately 2-year period. We found that our data were consistent with the trends that have been identified in other studies outside the United States, in which the prevalence of Salmonella in dogs and cats has declined significantly since the 1980s. This decline, in part, has been attributed to tighter food safety controls at the factory level and an increase in feeding commercially produced food instead of scraps (65, 66, 104). Additionally, this report confirms the association between feeding dogs raw foods and being infected with Salmonella. Since almost half of the infected animals do not have clinical signs, the owners may not be aware of the risk of becoming infected themselves.

The present study provides some recent baseline data of the Salmonella prevalence in pets in multiple states, data that can be used when investigating Salmonella outbreaks. In addition, the present study provides both PFGE and susceptibility patterns. We provide complete sequence data of the pathogens isolated during the study period, which is unique to this study, to facilitate future outbreak investigations.

## MATERIALS AND METHODS

### Study design.

Eleven collaborating laboratories participated in the Collaborative Laboratory Agreement Salmonella Project (V-CLASP), a Cooperative Agreement U-18-funded study organized by the Veterinary Laboratory Investigation and Response Network (Vet-LIRN) at the Center for Veterinary Medicine (CVM) of the Food and Drug Administration. The initial goal was for each laboratory to collect and test 200 fecal samples from 100 nondiarrheic and 100 diarrheic dogs. Cats were also recruited for the study, but a specific number was not requested, as projected caseloads varied greatly among participating laboratories. All animals enrolled in the study were from households (client-owned animals). Some of the funded laboratories also obtained samples from satellite clinics or local veterinary partners that routinely refer cases to the institution. Clients were provided a brochure which described the study objectives and methods to be used. All laboratories obtained client consent approvals and Institutional Animal Care and Use Committee approval as required by their individual institutions.

### Case definition and participant characteristics.

A common procedure was established to recruit diarrheic and nondiarrheic patients (dogs and cats) for the study. A diarrheic patient was defined as “an animal presented by owner to a veterinarian with a current problem of diarrhea.” Only one animal per household could be enrolled in the study. The V-CLASP group developed pamphlets describing the study to share with owners. Each institution followed its own procedures to document client consent for participation in the study. At the time of sample collection, owners answered a standardized questionnaire about the pet's diet, health status, current drug therapy, living environment, and outside exposures (S3-Questionnaire). Data were submitted electronically to the Vet-LIRN Program Office (VPO), which maintained a master data spreadsheet. All laboratories performed a final audit of their data. Discrepancies were investigated, and corrections were documented.

### Temperature data.

Due to the wide variation in temperatures in the different states in a given month, we used the average monthly temperature reported in the state where the fecal sample was collected (http://www.ncdc.noaa.gov/cag/) to evaluate potential effects of temperature. Other websites report daily temperatures; however, the data have not been quality controlled (https://www.ncdc.noaa.gov/temp-and-precip/asos/), and therefore, we chose the monthly temperature.

### Fecal collection and bacteriologic analysis.

Fecal samples were collected between January 2012 and April 2014. Samples were collected from clinical cases visiting the V-CLASP institution or from veterinarians at participating referral practices. Some samples were obtained by the veterinarian directly from the animal's rectum. Other samples were brought to the clinic by owners who had collected the fecal sample immediately after defecation. Most cat samples were retrieved in the morning from litter boxes that had been cleaned the night before. Samples were refrigerated and taken to the veterinarian the same day, preferably within 6 h. Samples obtained by local, collaborating veterinary clinics were shipped to the corresponding V-CLASP laboratory on ice packs, and samples were refrigerated upon arrival. Samples were processed within 24 h of arrival. A minimum of 1 g was required, but most samples collected were >10 g.

Samples were cultured for Salmonella using a harmonized method developed by the V-CLASP laboratories based on ISO 6579:2002 Annex D “Detection of Salmonella spp in animal feces and in samples of the primary production stage” (105) with several modifications, including the use of Rappaport-Vassiliadis (RV) broth instead of modified semisolid Rappaport Vassiliadis (MSRV) for enrichment and the use of xylose-lysine-tergitol 4 (XLT4) and brilliant green with novobiocin (BGN) instead of xylose lysine deoxycholate (XLD) for the selective medium. Media in identical lots were obtained from the same vendor. In short, 10 g of fecal sample was inoculated in Whirl-Pak bags (Nasco, California, USA) containing 90 ml of buffered peptone water (BPW) (Remel, Kansas, USA), and bags were gently massaged and then incubated at 37°C ± 1°C for 18 h ± 2 h. If 10 g of fecal sample was not available, then a 1:10 (wt/vol) dilution with BPW was performed. A 0.1-ml aliquot of the BPW-fecal mixture was incubated in 10 ml of RV broth (Remel) at 41.5°C ± 1°C for 24 h ± 3 h. Next, XLT4 agar (Remel) and BGN agar (Remel) were inoculated with 10 μl of sample and incubated, inverted, at 37°C for 24 h ± 3 h. Presumptive Salmonella colonies (at least 5, or all if less than 5) were selected for confirmation by plating them on MacConkey agar (Remel) and incubating them, inverted, at 37°C ± 1°C for 24 h ± 3 h. Biochemical testing was done by inoculation into triple sugar iron (TSI) agar (Remel) and lysine iron agar (LIA) (Remel) tubes and incubation at 37°C ± 1°C for 24 h ± 3 h. Alterations in the method were recorded and submitted by the laboratories on the questionnaire. Salmonella O antisera were used to confirm the presence of Salmonella (Poly A-I + Vi latex agglutination kit; Becton Dickinson and Co., Sparks, MD, USA). Positive isolates were cultured overnight at 37°C on Trypticase soy agar (TSA) with 5% sheep blood, and a single colony was used to inoculate a TSA tube, which was cultured overnight at 37°C. The TSA tube was submitted to the National Veterinary Services Laboratories (NVSL; Ames, IA, USA) for serotyping. Isolates were also submitted to the VPO for further testing and archiving. The VPO submitted additional blinded samples to the NVSL to confirm serovars. Positive cases were reported to the collaborating clinical veterinarian, and follow-up fecal cultures were offered at no cost to the owners; however, results from follow-up cases were not included in the statistical analysis.

### Proficiency tests.

During the study, V-CLASP laboratories participated in at least three Salmonella proficiency tests. These tests were part of the routine proficiency testing program administered by Vet-LIRN in collaboration with the Center for Food Safety and Applied Nutrition's Division of Food Processing Science and Technology at the Institute for Food Safety and Health Illinois Institute of Technology.

### AST.

Frozen isolates were streaked onto TSA with 5% sheep blood and incubated overnight at 35°C. Suspensions equivalent to a 0.5 McFarland standard were prepared and adjusted to 1.0 × 10^5^ CFU/ml in cation-adjusted Mueller-Hinton broth. This suspension was then used to inoculate the dehydrated panel format described below (provided by Trek Diagnostics, ThermoFisher Scientific, Cleveland, OH), which was incubated at 35°C for 18 h. We used the COMPAN2F panel designed for testing pathogens isolated from small animals. Broth microdilution testing was performed against amikacin, amoxicillin-clavulanic acid (2:1 ratio), ampicillin, cefazolin, cefovecin, cefoxitin, cefpodoxime, ceftiofur, cephalothin, chloramphenicol, clindamycin, doxycycline, enrofloxacin, erythromycin, gentamicin, imipenem, marbofloxacin, oxacillin plus 2% NaCl, penicillin, rifampin, ticarcillin-clavulanic acid constant 2, ticarcillin, and trimethoprim-sulfamethoxazole, in accordance with the Clinical and Laboratory Standards Institute (CLSI) standard guidelines VET01-A4 (106) and M100-S22 (107). Sensititre NARMS Gram-negative panels (CMV2AGNF and CMV3AGNF) (Trek Diagnostics, ThermoFisher Scientific, Cleveland, OH) were used on a subset of isolates to confirm the findings. The CMV3AGNF panels replaced the discontinued CMV2AGNF panels during the study. The CMV3AGNF panel does not have kanamycin, and there are more streptomycin concentrations. Broth microdilution testing on the Gram-negative panel was performed against amoxicillin-clavulanic acid (2:1 ratio), ampicillin, azithromycin, cefoxitin, ceftriaxone, ceftiofur, ciprofloxacin, chloramphenicol, gentamicin, kanamycin, nalidixic acid, streptomycin, sulfisoxazole, tetracycline, and trimethoprim-sulfamethoxazole.

Veterinary breakpoints were used when available to attribute sensitive or resistant status for the following drugs: amikacin (≥64 mg/liter), amoxicillin-clavulanic acid (2:1 ratio) (≥32/16 mg/liter), ampicillin (≥32 mg/liter), cefazolin (≥32 mg/liter), cephalothin (≥32 mg/liter), chloramphenicol (≥32 mg/liter), enrofloxacin (≥4 mg/liter), gentamicin (≥8 mg/liter), imipenem (≥16 mg/liter), marbofloxacin (≥4 mg/liter), sulfisoxazole (≥512 mg/liter), tetracycline (≥16 mg/liter), ticarcillin-clavulanic acid constant 2 (≥128/2 mg/liter), and trimethoprim-sulfamethoxazole (≥4/76 mg/liter) (108, 109). In the absence of veterinary breakpoints, human breakpoints were used for cefoxitin (≥32 mg/liter), cefpodoxime (≥8 mg/liter), ceftriaxone (≥4 mg/liter), ciprofloxacin (≥1 mg/liter), doxycycline (≥16 mg/liter), kanamycin (≥64 mg/liter), nalidixic acid (≥32 mg/liter), and ticarcillin (≥128 mg/liter) (110). NARMS breakpoints were used for azithromycin (≥32 mg/liter), ceftiofur (≥8 mg/liter), and streptomycin (≥64 mg/liter); the breakpoint for cefovecin (≥32 mg/liter) was used per the package insert. Intermediate values were classified as sensitive in our analysis.

### Pulsed-field gel electrophoresis (PFGE).

Frozen isolates were streaked onto TSA with 5% sheep blood for PFGE and whole-genome sequencing. PFGE was completed in accordance with CDC PNL05 (111).

### Whole-genome sequencing (WGS).

The same isolate used for PFGE was grown on TSA with 5% sheep blood agar plates prior to DNA extraction using a DNeasy blood and tissue kit (Qiagen, CA, USA). DNA was quantified using Qubit dsDNA BR assay kits (catalog no. Q21850; Life Technologies, NY, USA). WGS was performed on the MiSeq platform with paired-end 2 × 300-bp reads using v3 reagent kits. Reads were assembled *de novo* using CLC Genomics Workbench version 8.0 with automated assembly parameters. Each genome had at least 20-fold coverage, with N50 values of >30 kb.

### PCR comparison.

Nine laboratories conducted an optional evaluation of their in-house PCR methods for detecting Salmonella in dog feces by testing archived frozen samples in BPW. Four of the laboratories used the same method, i.e., MicroSEQ, while the other five laboratories used individual in-house methods. Some of the samples had been frozen for approximately 1.5 years. Culture-positive samples were included as test samples. Culture-negative samples from a different submission were used as negative controls for the study. There were many more negative samples, and therefore we selected the sample that had been submitted to the laboratory just prior to the submission of the culture-positive sample. The positive and time-matched negative samples were shipped to VPO by each collaborating laboratory. Participants received 34 previously culture-positive samples and 34 negative blinded samples to test. The 34 Salmonella-positive isolates comprised 13 different serovars. Of the previously culture-positive samples, only 13 could be reisolated from the old BPW sample and 21 samples had become culture negative. All 34 previously positive samples were used for this comparison regardless of the ability to reisolate the bacteria.

### Statistical analysis.

All statistical analyses were conducted using SAS/STAT software, version 9.3, of the SAS System for Windows (SAS Institute, Inc., Cary, NC, USA).

The data set was analyzed for statistically significant associations between the Salmonella status and other factors in univariate analysis, and Fisher's exact test was performed to test for an association at the 5% significance level. Multiple logistic regression analysis was conducted to evaluate the association between the Salmonella status and factors of interest after controlling for other factors. Odds ratios (OR) and 95% confidence intervals (CI) were calculated where appropriate.

If the status of any factor was reported missing or unknown, then the animal was excluded from the analysis. Therefore, a total of 62 dogs were excluded from the Salmonella-versus-probiotic status analysis because of “unknown” probiotic status. One dog with unknown probiotic status was Salmonella positive. Similarly, a total of 12 dogs were excluded from the Salmonella-versus-indoor/outdoor status analysis because data were missing or unknown. A total of 57 dogs were excluded from the Salmonella-versus-antibiotic use analysis. All of these dogs were Salmonella negative. Similarly, 113 dogs were excluded from the analysis when evaluating whether diarrhea status was affected by antibiotic use, because data were missing on one or both of these factors. Only two of the excluded dogs were Salmonella positive.

A total of 46 dogs were excluded from the Salmonella-versus-residential status analysis. Of these, 33 dogs had unknown residence status and were therefore excluded. These 33 dogs were all Salmonella negative. The remaining 13 dogs lived in multiple residential locations, and thus they were also excluded from the analysis. One multiple-residence-status dog was Salmonella positive, while the other 12 multiple-residence dogs were Salmonella negative.

Statistical analysis was not conducted on the cat data, because only three cats were Salmonella positive.

### Accession number(s).

Whole-genome sequences of each isolate were submitted to NCBI under BioProject accession no. PRJNA314600 (Vet-LIRN V-CLASP-Salmonella enterica genome sequencing). Resistance genotypes were determined by using CVM's resistance gene database, performing BLASTx analysis to identify resistance genes with at least 85% identity and 50% length relative to those in the database (112). SNPs were identified through the NCBI Pathogen Detection Isolates Browser (113).

## Supplementary Material

Supplemental material
