# Supplementary material for: Multilaboratory Survey To Evaluate Salmonella Prevalence in Diarrheic and Nondiarrheic Dogs and Cats in the United States between 2012 and 2014
Source: J Clin Microbiol. 2017 Apr 25;55(5):1350–68. doi: 10.1128/JCM.02137-16 (PMC5405253; doi:10.1128/JCM.02137-16)
Supplement: Supplemental material [file supp_55_5_1350__index.html]

Multilaboratory Survey To Evaluate Salmonella Prevalence in Diarrheic and Nondiarrheic Dogs and Cats in the United States between 2012 and 2014 — Supplemental material 

# Multilaboratory Survey To Evaluate Salmonella Prevalence in Diarrheic and Nondiarrheic Dogs and Cats in the United States between 2012 and 2014

## Supplemental material

- Supplemental file 1 -

  Table S1 (Studies of *Salmonella* prevalence in dogs)

  PDF, 133K
- Supplemental file 2 -

  Table S2 (Studies of *Salmonella* prevalence in cats)

  PDF, 52K
- Supplemental file 3 -

  Questionaire S3 (V-CLASP questionaire)

  PDF, 40K
- Supplemental file 4 -

  Table S4 (Comparision of antibacterial activity results versus those predicted by genotype)

  PDF, 117K
