# Supplementary material for: Multilaboratory Survey To Evaluate Salmonella Prevalence in Diarrheic and Nondiarrheic Dogs and Cats in the United States between 2012 and 2014
Source: J Clin Microbiol. 2017 Apr 25;55(5):1350–68. doi: 10.1128/JCM.02137-16 (PMC5405253; doi:10.1128/JCM.02137-16)
Supplement: Supplemental material [file JCM.02137-16_zjm999095450s1.pdf]

REF	Author	Year	Country	Animals	# dogs	% preva-lance	Diarrhea Healthy	Comments
(1)	Adesiyun	1997	Trinidad	all	130	4.6	DH	
	Adesiyun	1997	Trinidad	clinic	65	6.2	D	
	Adesiyun	1997	Trinidad	clinic	65	3.1	H	
(2)	Adler	1951	USA	kennel-quarantine	295	13.2		Hawaii
(3)	Amtsberg	1979	Germany	clinic	1246	9.1		Necropsy and sick animals, between 1974-1978, more in fecal (9.1) than organs (8%)
(4)	Bagcigil	2007	Turkey	all	200	1	DH	One dog dirrahea, the others healthy
	Bagcigil	2007	Turkey	clinic	100	1		
	Bagcigil	2007	Turkey	kennel	100	1		
(5)	Ball	1951	Bermuda	clinic	41	2.4		
	Ball	1951	Bermuda, USA	all	300	5.7		
	Ball	1951	USA	clinic, shelter	259	6.2		Los Angeles
(6)	Boargob	1975	Germany	all	312	10.3		Dissertation, Secondary source: Boos 1977
	Boargob	1975	Germany	clinic	77	9.9		
	Boargob	1975	Germany	pet shop	130	12.3		
	Boargob	1975	Germany	stray	105	8.6		
(7)	Boos	1977	Germany	all	533	16.6		Reported percent is of samples, not dogs (171 positive of 1003 samples)
	Boos	1977	Germany	clinic	25	0		
	Boos	1977	Germany	kennel-dog school	399	10.8		Reported percent is of samples, not dogs. (528 samples)
	Boos	1977	Germany	kennel-military	109	25.2		Reported percent is of samples not dogs. Guard dogs, 10 different groups sampled (repeated sampling-up to 4 times, total 480 samples)
(8)	Britt	1978	Nigeria	clinic	120	18		
(9)	Butler	1965	USA	all	173	9.2		Alaska, Central
	Butler	1965	USA	clinic	98	16.3		Alaska, Household pets (4 dogs had repeated sampling)
	Butler	1965	USA	kennel	75	0		Alaska, Sled dogs, 19 had repeated sampling
(10)	Butler	1967	USA	all	132	6.1	DH	Alaska, Fairbanks, one kennel and one military dog had diarrhea, the rest were healthy
	Butler	1967	USA	clinic	29	24	H	Alaska, Fairbanks, Household pets (4 dogs had repeated sampling)
	Butler	1967	USA	clinic	51	0	H	Alaska, Military base, Household pets
	Butler	1967	USA	kennel	25	0	H	Alaska, Private kennel
	Butler	1967	USA	kennel-military	27	3.7	H	Alaska, 24 dogs repeated sampling
(11)	Cantor	1997	USA	all	79	63	DH	Iditarod Trail Sled 1996, healthy plus diarrhea, before and during race, repeated sampling
	Cantor	1997	USA	sled dogs	30	63	D	Iditarod Trail Sled 1996, diarrhea during race, repeated sampling
	Cantor	1997	USA	sled dogs	26	69	H	Iditarod Trail Sled 1996, healthy before race, repeated sampling
	Cantor	1997	USA	sled dogs	23	57	H	Iditarod Trail Sled 1996, healthy during race, repeated sampling
(12)	Caraway	1959	USA	kennel-military	23	78.2		Lousiana, Outbreak
(13)	Cardaras	1985	Italy	clinic	24	0		
(14)	Cave	2002	USA	all	332	0.9	DH	
	Cave	2002	USA	clinic	260	1.2	D	
	Cave	2002	USA	clinic	72	0	H	
(15)	Corradini	1979	Italy	clinic	2138	7.16		
(16)	Cruickshank	1949	England	shelter	500	1		

REF	Author	Year	Country	Animals	# dogs	% preva-lance	Diarrhea Healthy	Comments
(17)	Day	1963	USA	laboratory	15	100		Lab exposure: single dose, all shed salmonella sporadically, no diarrhea, most stools positive days 4-5. Repeated sampling
(18)	Finley	2007	Canada	laboratory	16	43.8		Lab exposure: 16 dogs fed salmonella contaminated raw food - 7 shed Sal 1-7 days later, no clinical signs
(19)	Forster	1974	Germany	all	307	18		Necropsy specimens at Vet-Univeristy pathology lab
	Forster	1974	Germany	clinic	192	25		Dogs less than 6 months old
	Forster	1974	Germany	clinic	97	5.2		Dogs older than 6 months old
(20)	Frost	1969	Australia	all	305	6.9		
	Frost	1969	Australia	clinic	157	4.5	H	
	Frost	1969	Australia	shelter	138	8.7	H	
	Frost	1969	Australia	working	10	20	H	Dogs worked at abattoir
(21)	Fukata	2002	Japan	clinic	1013	0.1	H	Samples from fecal or lymph nodes: 0.1% fecal, nodes=1 of 8 (12.5%)
(22)	Fukushima	1985	Japan	shelter	252	15.5		Samples from fecal or lymph nodes: More positives in nodes than feces
(23)	Galton	1952	USA	all	8157	27.6		2252 total dogs positive, (repeated sampling for 524 dogs of which were 88% pos.)
(24)	Gorham	1951	USA	clinic	809	1		
(25)	Goudswaard	1969	Netherlands	unknown	300	11.3		
(26)	Hackett	2003	USA	all	130	2.3	DH	Colorado, most healthy, 12 dogs tested with diarrhea
	Hackett	2003	USA	clinic	71	2.8	D	
	Hackett	2003	USA	clinic	59	1.7	H	
(27)	Holland	1978	Germany	clinic	671	15.4		
(28)	Holt	1980	England	all	100	1	DH	
	Holt	1980	England	clinic	50	0	D	
	Holt	1980	England	clinic	50	0.5	H	
(29)	Jajere	2014	Nigeria	clinic, household	119	43.7		
(30)	Jay-Russell	2014	USA-Mexico	shelter, agriculture land	358	9.2		Arizona, Yuma-shelter dogs, California, Imperiala Valley, 103 coyote (32%)-fecal from ground near agricultural land
(31)	Joffe	2002	Canada	all	20	15		Different foods: 10 fed commercal dry- 0 pos, 10 fed raw diet that was 80% positive for Sal.
	Joffe	2002	Canada	household	10	0		fed commercial dry food, no raw food
	Joffe	2002	Canada	household	10	30		fed raw diet food that was 80% positivefor Salmonella
(32)	Kahn	1970	Sudan	all	442	23.5		
	Kahn	1970	Sudan	clinic	88	12.5		Khartoum Province
	Kahn	1970	Sudan	stray	304	28.3		Khartoum province, shot by police, necropsy/lymphnodes (26%) more from nodes than feces (5.3%)
	Kahn	1970	Sudan	stray	50	14		Upper Nile region, shot by police, necropsy
(33)	Kallo	2001	Iraq	clinic	150	11.3	DH	
(34)	Keyhani	1978	Iran	clinic	314	2.3		Owner of 1 of the 8 pos dogs also pos
(35)	Kintner	1949	USA	clinic	71	18		Ohio
(36)	Kocabiyik	2006	Turkey	shelter	82	11		Dogs had been in shelter 4-8 wks, 60% of dogs were healthy
(37)	Koopman	1972	Netherlands	laboratory - purchased	153	8.5	H	
(38)	Koopman	1973	Netherlands	laboratory - purchased	544	16.4	H	Some dogs multiple smaples- 89 positive, 23 pos at 1 week, 2 pos at 2 weeks, 2 dogs and 4 cats pos at 3 weeks.
(39)	Kozak	2003	Slovakia	clinic	187	0.53		Dogs presented with GI signs. 1 positive dog, inapppetent but no diarrhea

REF	Author	Year	Country	Animals	# dogs	% preva-lance	Diarrhea Healthy	Comments
(40)	Kunstyr	1962	Czechoslovakia	clinic	1147	0.95		Fecal samples 0.95% positive, necropsy organs from 671 dogs = 2.8% positive in 3 year period
(41)	Kwaga	1989	Nigeria	clinic	303	1	H	
(42)	Lauder	1974	Scotland	unknown	113	1		One positive animal- dog or cat not specified, prevalence estimated at 1 (worst case), Secondary source: Borland 1975
(43)	Lefebvre	2008	Canada	all	194	2.6		Therapy dogs, reporting baseline. incidence rate 0.61 cases/dog-year raw meat fed vs 0.08 cases/dog-year not fed raw meat
	Lefebvre	2008	Canada	household	154	2.6		Therapy dogs, no raw food, point prevalance at varous times 0-2.6
	Lefebvre	2008	Canada	household	40	25		Therapy, ate raw food point prevalance at varous times 2.5-25
(44)	Lenz	2009	USA	all	91	6.6	H	
	Lenz	2009	USA	household	42	14.3	H	Ohio, ate raw food
	Lenz	2009	USA	household	49	0	H	Ohio, ate no raw food
(45)	Leonard	2011	Canada	household	138	23		Repeated sampling, 14 of 32 positive dogs ate raw food
(46)	Lowden	2015	England	all	436	0.2	H	
	Lowden	2015	England	household	126	0.8	H	
	Lowden	2015	England	kennel- retired racing greyhound	39	0	H	
	Lowden	2015	England	kennel- boarding	43	0	H	
	Lowden	2015	England	laboratory	132	0	H	
	Lowden	2015	England	shelter	96	0	H	
(47)	Mackel	1952	USA	household	1626	15		Florida, Short carrier state (also reported in Galton, #4 in series)
(48)	Mann	1969	USA	shelter	200	2		
(49)	Matsumoto	1966	Japan	shelter	1341	14	H	Data mainly from lymph nodes cultures but some fecal samples included, apparently healthy.
(50)	Mayer	1976	Germany	working-military dogs	67	41.5		Outbreak, police dogs fed raw food Feb 1974, second exam 54.6% positive, May (after stopping raw food) 15% positive
(51)	McElrath	1952	USA	all	3459	17.9		Florida, (also reported in Galton, #3 in series)
	McElrath	1952	USA	clinic	2438	21.5		Florida, repeated sampling (also reported in Galton, #3 in series)
	McElrath	1952	USA	kennel	126	16.6	H	Florida, (also reported in Galton, #3 in series)
	McElrath	1952	USA	shelter	895	7.8		Florida, (also reported in Galton, #3 in series)
(52)	McKenzie	2010	USA	all	135	74		Iditarod Trail Sled 2008, before and during race, repeated sampling, diarrhea in 12% (6/49) before race, diarrhea in 36% during race. Salmonella prevalence decreased from 78 to 71.
	McKenzie	2010	USA	sled dogs	55	78		Iditarod Trail Sled 2008, before race-diarrhea in 12% (6/49)
	McKenzie	2010	USA	sled dogs	80	71		Iditarod Trail Sled 2008, after 400 miles-during race, diarrhea in 36%, (29/80)
(53)	Moreley	2006	USA	kennel-racing greyhounds breeding	61	93		Colorado, outbreak, fecal samples collected from floor, pups were most affected
(54)	Mortelmans	1961	Brundi	unknown	190	3.7		
	Mortelmans	1961	Ruanda	unknown	682	1.6		
(55)	Murphy	2009	Canada	clinic	188	0		
(56)	Nastasi	1986	Italy	park	212	2.3		Fecals from ground

REF	Author	Year	Country	Animals	# dogs	% preva-lance	Diarrhea Healthy	Comments
(57)	Neill	1981	N. Ireland	all	127	3.9	DH	
	Neill	1981	N. Ireland	clinic	38	10.5	D	Necropsy samples
	Neill	1981	N. Ireland	clinic	66	1.5	D	Patients
	Neill	1981	N. Ireland	shelter	23	0	H	
(58)	Nilsson	1955	Sweden	all	215	1.9		Necropsy (158) or fecal exams (57). Only 7 positives in the necropsy group- 4 had clinical enteritis)
	Nilsson	1955	Sweden	clinic	158	4.4		Necropsy, 7 pos, 4 had enteritis
	Nilsson	1955	Sweden	clinic	57	0		Fecal exam, 0 pos, unknown health status
(59)	Ojo	1974	Nigeria	clinic	125	8	H	
(60)	Ojo	1994	Trinidad	stray	100	0		Necropsy intestinal contents, not lymph nodes. Feces was very dry on post mortem, may have affected results
(61)	Ojo	2009	Nigeria	all	458	3.7	DH	
	Ojo	2009	Nigeria	clinic, household	126	4	D	
	Ojo	2009	Nigeria	clinic, household	332	3.6	H	
(62)	Oosterom	1980	Netherlands	household, park	25	0.2		Positive fecals obtained from ground in park. Household dog fecal samples were negative
(63)	Osman	1970	Tunisia	all	1321	0.8		
	Osman	1970	Tunisia	household	1221	0.5		rural
	Osman	1970	Tunisia	household	100	4	H	urban
(64)	Polpakdee	2012	Thailand	all	500	12.4	DH	
	Polpakdee	2012	Thailand	clinic	250	11.6	D	
	Polpakdee	2012	Thailand	clinic	250	13.2	H	
(65)	Proctor	2014	Canada	park	251	1.2		Normal fecal samples collected from ground
(66)	Queisser	1970	Germany	working-military dogs	714	0.6	H	
(67)	Rioche	1960	Algiers	clinic	100	4	H	Necropsy
(68)	Schaffert	1978	Germany	park	300	10.3		Fecals from ground
(69)	Schotte	2007	Germany	kennel-military	80	63.8	DH	Outbreak, related to deydrated feed, 16.9% were symptomatic. Between 1996-2002 low prev, 0.6% routine testing 2x/yr, repeated sampling
(70)	Seepersadsingh	2004	Trinidad	all	1391	3.6	H	
	Seepersadsingh	2004	Trinidad	clinic	181	2.2	H	
	Seepersadsingh	2004	Trinidad	household	350	2.3	H	
	Seepersadsingh	2004	Trinidad	household-hunting	100	5	H	
	Seepersadsingh	2004	Trinidad	kennel-quarantine	125	11.2	H	
	Seepersadsingh	2004	Trinidad	pet shop	8	0	H	
	Seepersadsingh	2004	Trinidad	shelter	371	4.9	H	
	Seepersadsingh	2004	Trinidad	working-farm	110	0	H	
(71)	Selmi	2011	Italy	shelter	41	60.9	DH	Outbreak related to dehydrated feed, healthy and diarrhea dogs, repeated sampling, infection prevealance per sampling 12.5-34%), fecal samples were collected from the floor and pooled in some cases
(72)	Shimi	1976	Iran	all	672	7.7	H	
	Shimi	1976	Iran	household	472	4.4	H	
	Shimi	1976	Iran	kennel	181	15.5	H	
	Shimi	1976	Iran	stray	19	15.8	H	
(73)	Smith	1950	England	clinic	100	1		
(74)	Smith	1959	England	unknown	200	0.5		Necropsy (fecal 0.5%, necropsy samples: nodes 4.5 % positive)
(75)	Sokolow	2005	USA	shelter	120	0		California

REF	Author	Year	Country	Animals	# dogs	% preva-lance	Diarrhea Healthy	Comments
(76)	Stucker	1952	USA	kennel-racing greyhounds	572	67.7		Florida, Racing Greyhounds, 46.8% positive the first sampling, repeated sampling (also reported in Galton, #2 in series)
(77)	Sugiyama	1993	Japan	shelter	283	3.5		Noted recent arrivals higher incidence 12.2 %
(78)	Tanaka	1976	Japan	stray	100	44	H	Repeated sampling: series 1: 48 dogs, 17 pos (35.4%, 8 fecal positive- 17%) series 2: 52 dogs, 27 pos (51.9% , 22 fecal positive 46.8%)
(79)	Tesfamariam	1973	Germany	all	230	25.7		Secondary source: Borland 1975, Boos 1977
	Tesfamariam	1973	Germany	clinic	71	18.3		Necrospy, Secondary source: Boos 1977
	Tesfamariam	1973	Germany	pet shop	84	41.7		Secondary source: Boos 1977
	Tesfamariam	1973	Germany	stray	75	14.6		Secondary source: Boos 1977
(80)	Timbs	1975	New Zealand	all	600	3		
	Timbs	1975	New Zealand	clinic	150	0	H	
	Timbs	1975	New Zealand	stray	150	3.3		
	Timbs	1975	New Zealand	working-farm	150	4.7		Dogs on farm with sheep known to have positive *Salmonella* in past 12 months
	Timbs	1975	New Zealand	working-farm	150	4		Dogs on farm with sheep negative for *Salmonella* in past 12 months
(81)	Topacio	1963	Philippines	All	269	0		
	Topacio	1963	Philippines	Clinic	212	0		
	Topacio	1963	Philippines	Pound	57	0		
(82)	Tsai	2007	Taiwan	all	928	4.3		
	Tsai	2007	Taiwan	clinic	437	2.1	DH	13 dogs with diarrhea, only was 1 positive
	Tsai	2007	Taiwan	shelter	491	6.3		
(83)	Tupler	2012	USA	all	100	4		Florida
	Tupler	2012	USA	shelter	50	2	D	
	Tupler	2012	USA	shelter	50	6	H	
(84)	Van der Gulden	1970	Netherlands	laboratory	340	1.8		
(85)	Van Duijkeren	2002	Netherlands	clinic	6589	1	D	Data from lab database-1993-2002. 69 fecals positive, 11 positives in other samples
(86)	Van Schothorst	1978	Netherlands	household	49	1		Dogs from household with salmonella infected infants, 5 households had salmonella positive dog food in 31 samples.
(87)	Varela	1951	Mexico	kennel-quarantine	100	9		
(88)	Venter	1988	South Africa	clinic		9.5		Number of animals not given, clinical cases, poor community, run free at night, poor body condition, most less than 1 yr old
(89)	Verma	2011	India	clinic	250	0.4	H	1 positive fecal sample, 2 positive blood samples
(90)	Watt	1950	USA	household	1156	3.4		Texas, survey done near infected humans
(91)	Weber	1995	Germany	clinic, household	2985	3.5		Data from lab database-1975-1994 from vets or private owners, animals with GI signs
(92)	Wolff	1948	USA	all	74	8.1		Michigan, 46 dogs ill, 6 were positive (13%), healthy dogs - boarding or stray were all neg (non-outbreak),
	Wolff	1948	USA	clinic	46	13		Michigan, 46 dogs ill, 6 were positive (13%)
	Wolff	1948	USA	kennel	27	48.1		Michigan, outbreak, repeated sampling, "Kennel" single room with 25-30 dogs, often sick, 13 of 27 dogs positive (48.1%)
	Wolff	1948	USA	shelter	28	0	H	Michigan, healthy dogs - boarding or stray were all neg
(93)	Ximenes	1981	Italy	clinic	150	6.7	DH	Healthy and ill dogs
(94)	Zenad	2003	Iraq	stray	40	15		
(95)	Zwart	1962	Ghana	unknown	60	8.3		
