# Supplementary material for: Multilaboratory Survey To Evaluate Salmonella Prevalence in Diarrheic and Nondiarrheic Dogs and Cats in the United States between 2012 and 2014
Source: J Clin Microbiol. 2017 Apr 25;55(5):1350–68. doi: 10.1128/JCM.02137-16 (PMC5405253; doi:10.1128/JCM.02137-16)
Supplement: Supplemental material [file JCM.02137-16_zjm999095450s2.pdf]

REF	Author	Year	Country	Animals	# cats	% preva-lance	Diarrhea Healthy	Comments
(1)	Amtsberg	1979	Germany	clinic	480	8.4		Fecal 8.4%, organs 10.1%
(2)	Ball	1951	USA	clinic, shelter	200	2		
(3)	Cardaras	1985	Italy	clinic	2	0		
(4)	Cruickshank	1949	England	clinic, shelter	500	1.4		Necropsy- unwanted cats
(5)	Fox	1979	USA	laboratory	142	10.6		Massachusetts,random source, repeat sampling, 3 rectal samples each cat
(6)	Fukushima	1985	Japan	shelter	318	2.8		
(7)	Gorham	1951	USA	clinic	91	0		
(8)	Hill	2000	USA	all	199	1	DH	Colorado
	Hill	2000	USA	household	70	1.4	D	Colorado
	Hill	2000	USA	household	52	0	H	Colorado
	Hill	2000	USA	shelter	12	0	D	Colorado
	Hill	2000	USA	shelter	53	1.9	H	Colorado
	Hill	2000	USA	shelter	12	0		Colorado, unknown health status
(9)	Kahn	1970	Sudan	clinic	19	10.5		Necropsy- unwanted cats
(10)	Koopman	1972	Netherlands	laboratory	149	4.7		Lab exposure: cats were treated with tetracycline, carrier state under lab conditions only about 1 week
(11)	Koopman	1973	Netherlands	laboratory - purchased	485	6.8		
(12)	Murphy	2009	Canada	clinic	39	0		
(13)	Oosterom	1980	Netherlands	household	10	0		Samples collected by owners
(14)	Polpakdee	2012	Thailand	all	100	9	DH	
	Polpakdee	2012	Thailand	clinic	50	10	H	
	Polpakdee	2012	Thailand	clinic	50	8	D	
(15)	Shimi	1977	Iran	all	301	13.6	DH	
	Shimi	1977	Iran	clinic	32	37.5	D	
	Shimi	1977	Iran	clinic	109	12.8	H	
	Shimi	1977	Iran	shelter	160	9.4	H	
(16)	Smith	1959	England	unknown	200	0.5		Fecal - 0.5%, lymph nodes-2.5%
(17)	Spain	2001	USA	all	263	0.8		New York
	Spain	2001	USA	household	114	0.9		New York
	Spain	2001	USA	shelter	149	0.7		New York
(18)	Van der Gulden	1970	Netherlands	laboratory	150	0.7		
(19)	Van Immerseel	2004	Belgium	all	371	6.5	DH	
	Van Immerseel	2004	Belgium	household	278	0.36	H	
	Van Immerseel	2004	Belgium	household	58	8.6	D	
	Van Immerseel	2004	Belgium	household	35	51.4	H	Kittens in group housing.
(20)	Watt	1950	USA	unknown	625	3		
(21)	Weber	1995	Germany	clinic, household	2024	1.9		
(22)	Zenad	2014	Iraq	stray	59	10.2	H	Rectal swabs and necropsy, captured strays, 13.5% overall positive with internal organs included
