# Supplementary material for: Multilaboratory Survey To Evaluate Salmonella Prevalence in Diarrheic and Nondiarrheic Dogs and Cats in the United States between 2012 and 2014
Source: J Clin Microbiol. 2017 Apr 25;55(5):1350–68. doi: 10.1128/JCM.02137-16 (PMC5405253; doi:10.1128/JCM.02137-16)
Supplement: Supplemental material [file JCM.02137-16_zjm999095450s3.pdf]

Supplement 3

Submission Form/Questionnaire-Medical & Diet History
Vet-LRN Cooperative Laboratory Agreement Salmonella Project

Date (mm/dd/yy)

Accession #: (for lab use only)

PATIENT INFORMATION

Owner Name: Last First

Address

City State Zip Code

County

Pet's Name

○ Dog ○ Cat

Breed

Age ☐ Years ☐ Months ☐ Days

Gender:

○ M ○ MN ○ F ○ FS

HISTORY

Reason for Veterinary Visit:

Previous Significant Medical History:

CLINICAL INFORMATION--Current Clinical Signs

Blood in Feces? ☐ Yes ☐ No Appearance of Blood: ☐ Fresh,Red ☐ Black,Tarry ☐ Coffee Ground

Diarrhea? ☐ Yes ☐ No

If Yes: Duration: in days

Was a fecal culture performed? ☐ Yes ☐ No

If a culture was performed, was Salmonella isolated? ☐ Yes ☐ No

If No: Has pet had diarrhea in past 30 days? ☐ Yes ☐ No

If diarrhea in past 30 days, was there medical history/diagnosis to explain diarrhea?

Abdominal Pain? ☐ Yes ☐ No

Vomiting? ☐ Yes ☐ No

Lethargy? ☐ Yes ☐ No

Other? ☐ Yes ☐ No Describe:

MEDICATIONS

Is your pet currently taking any medication? ☐ Yes ☐ No

If yes, what type?

Is your pet currently taking any probiotics? ☐ Yes ☐ No

If yes, what type?

Has your pet received antibiotics in the past month? ☐ Yes ☐ No

If yes, what type?

Continued other side

Questionnaire-Medical History
Vet-LRN Cooperative Laboratory Agreement Salmonella Project

Accession #: (for lab use only)

Owner:

Pet's Name:

DIET Please indicate pet's diet: check all that apply

☐ Commercial Dry — Indicate contribution to diet: ☐ Primary ☐ Secondary ☐ Occasional

List name or brands fed in last 30 days:

☐ Commercial Wet-Canned — Indicate contribution to diet: ☐ Primary ☐ Secondary ☐ Occasional

List name or brands fed in last 30 days:

☐ Commercial Wet-Pouch — Indicate contribution to diet: ☐ Primary ☐ Secondary ☐ Occasional

List name or brands fed in last 30 days:

☐ Commercial-Raw — Indicate contribution to diet: ☐ Primary ☐ Secondary ☐ Occasional

List name or brands fed in last 30 days:

☐ Homemade-Raw — Indicate contribution to diet: ☐ Primary ☐ Secondary ☐ Occasional

Specify ingredients

☐ Homemade-Cooked — Indicate contribution to diet: ☐ Primary ☐ Secondary ☐ Occasional

Specify Ingredients

☐ Table Scraps — Indicate contribution to diet: ☐ Primary ☐ Secondary ☐ Occasional

☐ Commercial-Treats — Indicate contribution to diet: ☐ Primary ☐ Secondary ☐ Occasional

List name or brands fed in last 30 days:

☐ Rawhide Treats — Indicate contribution to diet: ☐ Primary ☐ Secondary ☐ Occasional

☐ Chicken Jerky — Indicate contribution to diet: ☐ Primary ☐ Secondary ☐ Occasional

☐ Pig Ears — Indicate contribution to diet: ☐ Primary ☐ Secondary ☐ Occasional

☐ Food or treats other than above — Indicate contribution to diet: ☐ Primary ☐ Secondary ☐ Occasional

Describe:

☐ Outside Material (grass, carrion, rodents) — Indicate contribution to diet: ☐ Primary ☐ Secondary ☐ Occasional

Describe:

Continued other side

Submission Form/Questionnaire-Medical & Diet History
Vet-LRN Cooperative Laboratory Agreement Salmonella Project

Accession #: (for lab use only) ____

Owner: ____ Pet's Name: ____

EXPOSURES

Is your pet primarily: ☐ Indoor ☐ Outdoor ☐ Both

Is your pet used for hunting? ☐ Yes ☐ No If yes, type: ____

Is your pet a show pet? ☐ Yes ☐ No

Does your pet participate in pet sporting events or competitions? ☐ Yes ☐ No

Does your pet visit community pet recreation facilities, such as dog parks? ☐ Yes ☐ No

Is your pet exposed to: (check all those that apply)

- ☐ Livestock List livestock: ____
- ☐ Poultry List Poultry: ____
- ☐ Reptiles List reptiles: ____
- ☐ Pet Birds List birds ____
- ☐ Other List: ____

Is your pet exposed to untreated surface water (ponds, streams, etc.)? ☐ Yes ☐ No If yes, type? ____

HOUSEHOLD

How would you classify your pet's residence? ☐ Urban ☐ Suburban ☐ Rural

How many other pets inhabit household? (List number and type by age)

<1 year ____ 1- 5 years ____ 6-12 years ____

13-21 years ____ >21 years ____

Continued other side

Submission Form/Questionnaire-Medical & Diet History
Vet-LRN Cooperative Laboratory Agreement Salmonella Project

Owner: ________ Pet's Name: ________

FECAL SAMPLE COLLECTION (To be filled in by Veterinarian)

Collection Date: (mm/dd/yy) ________ ☐ Free Catch ☐ Digital ☐ Owner Collected ☐ Other

Time: (hh:mm) ________ ☐ AM ☐ PM If Other, Describe: ________

FOR LAB USE ONLY:

Accession #: ________ VCLASP Study Site: ________

Received Date: (mm/dd/yy) ________ Time: (hh:mm) ________ ☐ AM ☐ PM Received By: (initials) ________

Condition on Receipt: ☐ Acceptable ☐ Not Acceptable

Comments: ________

☐ Salmonella species Not Isolated ☐ Salmonella species Isolated

If Salmonella Isolated, Serogroup/Serotype ________

If Deviation from V-CLASP Study Protocol, Describe: ________

Submit Button
