# Supplementary material for: Multilaboratory Survey To Evaluate Salmonella Prevalence in Diarrheic and Nondiarrheic Dogs and Cats in the United States between 2012 and 2014
Source: J Clin Microbiol. 2017 Apr 25;55(5):1350–68. doi: 10.1128/JCM.02137-16 (PMC5405253; doi:10.1128/JCM.02137-16)
Supplement: Supplemental material [file JCM.02137-16_zjm999095450s4.pdf]

Table S4. Comparision of antibacterial activity result (MIC-µg/mL) vs predicted by genotype

Comment	Drug	Drug code	MIC break point µg/mL	D-01, *S*. Typhimurium			D-18, *S*. Derby			D-59, *S*. Albany			C-02, *S*. I 4,5,12: i:		
				Genotype WGS predicted	Phenotype COMPAN2F	Phenotype NARMs	Genotype WGS predicted	Phenotype COMPAN2F	Phenotype NARMs	Genotype WGS predicted	Phenotype COMPAN2F	Phenotype NARMs	Genotype WGS predicted	Phenotype COMPAN2F	Phenotype NARMs
a	Amikacin	AMI	≥64	S	≤ 4	-	S	≤ 4	-	S	≤ 4	-	S	≤ 4	-
a	Amoxicillin / clavulanic acid 2:1 ratio	AMC, AUG2	≥32/16	S	NI (> 1)	= 16	S	NI (> 1)	= 8	R	NI (> 1)	> 32	S	NI (> 1)	= 8
a	Ampicillin	AMP	≥32	R	NI (> 1)	> 32	R	NI (> 1)	> 32	R	NI (> 1)	> 32	R	NI (> 1)	> 32
d	Azithromycin	AZI	>32	S	-	= 4	S	-	= 4	S	-	= 4	S	-	= 4
a	Cefazolin	FAZ	≥32	S	= 4	-	S	= 4	-	R	NI (> 8)	-	S	= 4	-
e	Cefovecin	FOV	≥8	S	= 1	-	S	= 1	-	R	> 4[g]	-	S	= 0.5	-
c	Cefoxitin	FOX	≥32	S	≤ 2	= 2	S	= 4	= 4	R	> 16[g]	> 32	S	≤ 2	= 2
c	Cefpodoxime	POD	≥8	S	≤ 2	-	S	≤ 2	-	R	> 16	-	S	≤ 2	-
c	Ceftriaxone	AXO	≥4	S	-	≤ 0.25	S	-	≤ 0.25	R	-	= 16	S	-	≤ 0.25
d	Ceftiofur	XNL, TIO	≥8	S	= 1	= 1	S	= 1	= 1	R	> 4[g]	> 8	S	= 1	= 1
a	Cephalothin	CEP	≥32	S	= 4	-	S	= 8	-	S	NI (> 8)	-	S	= 8	-
a	Chloramphenicol	CHL	≥32	R	> 16[g]	> 32	S	= 8	= 8	S	= 8	= 4	S	≤ 4	= 8
c	Ciprofloxacin	CIP	≥1	S	-	≤ 0.015	S	-	≤ 0.015	S	-	≤ 0.015	S	-	0.03
c	Doxycycline	DOX	≥16	S	= 8	-	R	> 8[g]	-	S	≤ 2	-	S	≤ 2	-
a	Enrofloxacin	ENRO	≥4	S	= 0.5	-	S	≤ 0.25	-	S	≤ 0.25	-	S	≤ 0.25	-
a	Gentamicin	GEN	≥8	S	= 2	= 1	S	≤ 1	= 0.5	S	≤ 1	≤ 0.25	S	≤ 1	= 0.5
a	Imipenem	IMI	≥16	S	≤ 1	-	S	≤ 1	-	S	≤ 1	-	S	≤ 1	-
c	Kanamycin	KAN	≥64	R	-	> 64	R	-	> 64	S	-	-	S	-	-
a	Marbofloxacin	MAR	≥4	S	≤ 0.25	-	S	≤ 0.25	-	S	≤ 0.25	-	S	≤ 0.25	-
c	Nalidixic Acid	NAL	≥32	S	-	= 4	S	-	= 2	S	-	= 4	S	-	= 2
d	Streptomycin	STR	≥64	R	-	= 64	R	-	> 64	S	-	= 8	R	-	> 64
a	Sulfisoxazole	FIS	≥512	R	-	> 256[g]	R	-	> 256[g]	S	-	≤ 16	S	-	= 32
a	Tetracycline	TET	≥16	R	-	= 32	R	-	> 32	S	-	≤ 4	S	-	≤ 4
c	Ticarcillin	TIC	≥128	R	> 64[g]	-	R	> 64[g]	-	R	> 64[g]	-	R	> 64[g]	-
b	Ticarcillin / clavulanic acid constant 2	TIM2	≥128/2	S[f]	> 64[g]	-	S	= 64	-	R	> 64[g]	-	S	= 32	-
a	Trimethoprim / sulfamethoxazole	SXT, COT	≥4/76	S	≤ 0.5	= 0.25	S	≤ 0.5	≤ 0.12	S	≤ 0.5	≤ 0.12	S	≤ 0.5	≤ 0.12

[a] CLSI M31-A3 beakpoints used for interpretation
[b] CLSI Vet01-S2E beakpoints used for interpretation
[c] CLSI M100-S24 beakpoints used for interpretation
[d] NARMS beakpoints used for interpretation (http://www.fda.gov/downloads/AnimalVeterinary/SafetyHealth/AntimicrobialResistance/NationalAntimicrobialResistanceMonitoringSystem/UCM442212.pdf)
[e] EMA beakpoints used for interpretation (http://www.ema.europa.eu/docs/en_GB/document_library/EPAR_-_Product_Information/veterinary/000098/WC500062067.pdf)
[f] Discrepancy between phenotype and predicted genotype
[g] MIC is higher than highest drug concentration on the panel and reach the breakpoint
NI: No interpretation. Panel's highest drug concentration is lower than drugs' breakpoint
- : Drug is not available on that plate
